# Endoplasmic Reticulum Stress Impaired Uncoupling Protein 1 Expression via the Suppression of Peroxisome Proliferator-Activated Receptor γ Binding Activity in Mice Beige Adipocytes

**DOI:** 10.3390/ijms20020274

**Published:** 2019-01-11

**Authors:** Ana Yuliana, Asumi Daijo, Huei-Fen Jheng, Jungin Kwon, Wataru Nomura, Haruya Takahashi, Takeshi Ara, Teruo Kawada, Tsuyoshi Goto

**Affiliations:** 1 Laboratory of Molecular Function of Food, Division of Food Science and Biotechnology, Graduate School of Agriculture, Kyoto University, Gokasho, Uji, Kyoto 611-0011, Japan; anayulia@kais.kyoto-u.ac.jp (A.Y.); asumi.7.9.2.8@gmail.com (A.D.); ydnas@kais.kyoto-u.ac.jp (H.-F.J.); kwon.jungin.34r@st.kyoto-u.ac.jp (J.K.); nom2@kais.kyoto-u.ac.jp (W.N.); haruya@kais.kyoto-u.ac.jp (H.T.); ara@kais.kyoto-u.ac.jp (T.A.); fat@kais.kyoto-u.ac.jp (T.K.); 2 Research Unit for Physiological Chemistry, the Center for the Promotion of Interdisciplinary Education and Research, Kyoto University, Kyoto 606-8501, Japan

**Keywords:** beige adipocytes, endoplasmic reticulum stress, peroxisome proliferator-activated receptor γ, uncoupling protein 1

## Abstract

Endoplasmic reticulum (ER) homeostasis is critical in maintaining metabolic regulation. Once it is disrupted due to accumulated unfolded proteins, ER homeostasis is restored via activation of the unfolded protein response (UPR); hence, the UPR affects diverse physiological processes. However, how ER stress influences adipocyte functions is not well known. In this study, we investigated the effect of ER stress in thermogenic capacity of mice beige adipocytes. Here, we show that the expression of uncoupling protein 1 (*Ucp1*) involved in thermoregulation is severely suppressed under ER stress conditions (afflicted by tunicamycin) in inguinal white adipose tissue (IWAT) both in vitro and in vivo. Further investigation showed that extracellular signal-regulated kinase (ERK) and c-Jun N-terminal kinase (JNK) were both activated after ER stress stimulation and regulated the mRNA levels of *Ucp1* and peroxisome proliferator-activated receptor γ (*Pparγ*), which is known as a *Ucp1* transcriptional activator, in vitro and ex vivo. We also found that Pparγ protein was significantly degraded, reducing its recruitment to the *Ucp1* enhancer, thereby downregulating *Ucp1* expression. Additionally, only JNK inhibition, but not ERK, rescued the Pparγ protein. These findings provide novel insights into the regulatory effect of ER stress on *Ucp1* expression via Pparγ suppression in beige adipocytes.

## 1. Introduction

Adipose tissue has been broadly characterized as white and brown adipocytes through their distinct characteristics in lipid metabolism. White adipose tissue (WAT) functions as energy storage, while brown adipose tissue (BAT) dissipates energy as heat supported by its high oxidative capacity [1,2,3]. Recent findings show inducible brown-like white adipocytes, known as beige adipocytes. Beige adipocytes are developed within WAT in response to β-adrenergic receptor (β-AR) stimulation, termed as browning of WAT, and exhibit similar characteristic as BAT [2,4]. The transcription of uncoupling protein 1 (*Ucp1*) is tightly regulated during browning, thus it is often used as a browning marker of adipose tissue [1,5]. Interestingly, in addition to browning, beige adipocytes also show unique plasticity to undergo whitening (reversal of browning) [6,7,8].

The endoplasmic reticulum (ER) is a critical organelle in sensing and handling cellular nutrient required for normal cellular functions and survival [9]. Certain physiological and pathological factors such as nutrient deprivation, lipids, or increased synthesis of secretory proteins can induce cell stress and disrupt ER homeostasis by promoting unfolded protein overload [9]. Unfolded protein response (UPR) is activated as an adaptive response to restore ER activity and maintain protein quality to ensure proper protein synthesis, secretion, and correct folding of protein [10,11,12]. When ER stress is severe and/or prolonged, it can shift toward apoptosis (cell death), although the mechanism for this transition is not well understood [10,13,14,15]. ER stress has been linked to multiple disorders ranging from neurodegenerative diseases to metabolic disorders such as obesity, insulin resistance, type 2 diabetes, and chronic inflammation [9,10,16]. Metabolically active tissue including adipose tissue is not excluded from the ER stress outcomes [17,18,19]. However, the consequences of ER stress on adipocyte functions including thermogenic capacity are poorly understood.

In adipose tissue, obesity results in chronic stress and dysfunction caused by the increased demand of synthetic machinery [17]. Several studies have shown a significant activation of ER stress in high-fat-induced obesity [20,21,22]. In addition, both brown and beige adipocyte activation stimulated by cold exposure was impaired in obese adipose tissue [23]. Adversely, a decrease in adaptive thermogenesis has also been suggested as a contributing factor to obesity [6]. As obesity is characterized by diverse metabolic symptoms such as ER stress and inflammation, there is still no direct link about how ER stress itself could regulate *Ucp1* expression in beige adipocytes.

Previous study has shown that the activation of downstream signaling in ER stress, such as that involving inositol-requiring enzyme 1α (IRE1α) and X-box binding protein 1 (XBP1), was needed for *Ucp1* expression in BAT [16]. Further, the inactivation of ER stress in BAT, WAT, or macrophages resulted in an improved adaptive thermogenesis response [6,24], while treatment of chemical chaperones (ER stress inhibitor) could increase energy expenditure and activate browning of WAT [25,26,27]. These reports suggest a possible unknown regulatory mechanism of ER stress in the browning of WAT. In this study, we investigated the effect of ER stress stimulation on *Ucp1*, an adipocyte browning marker, in beige adipocytes.

## 2. Results

### 2.1. ER Stress Decreases Ucp1 mRNA Level in Beige Adipocytes

**Figure 1:**
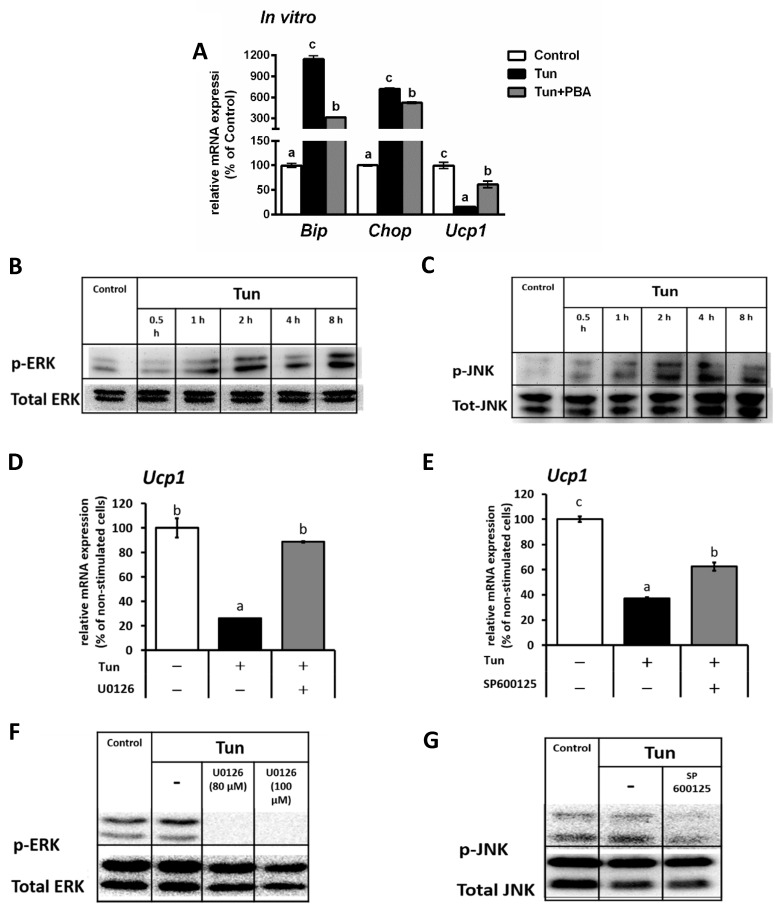
Endoplasmic reticulum (ER) stress stimulation downregulates uncoupling protein 1 (*Ucp1*) through phosphorylation of extracellular signal-regulated kinase (ERK) and c-Jun N-terminal kinase (JNK) in inguinal white adipose tissue (IWAT) cells. (**A**) the mRNA expression levels of ER stress markers: binding immunoglobulin protein (*Bip*), CCAAT-enhancer-binding protein homologous protein (*Chop*), and adipocyte browning markers *Ucp1* after treatment with 1 μM tunicamycin (Tun) for 12 h with or without 20 mM 4-phenylbutyrate (PBA) for 24 h before collection. Phosphorylation of (**B**) ERK and (**C**) JNK at different time points when treated with 1 μM tunicamycin. (**D**,**E**) *Ucp1* mRNA expression and phosphorylation of ERK (**F**) or JNK (**G**) after being treated with (**D**,**F**) 80 or 100 μM ERK inhibitor (U0126) for 14 h, or (**E**,**G**) 25 μM JNK inhibitor (SP600125) for 13 h, followed by tunicamycin (1 μM; 12 h). Data are presented as mean ± S.E.M. (error bars). *n* = 4 in each group. Different letters indicate significant differences (*p* < 0.05) according to one-way ANOVA followed by Tukey–Kramer multiple comparison test.

To investigate the regulation of ER stress on the thermogenic capacity of beige adipocytes, inguinal white adipose tissue (IWAT) cells were differentiated to beige adipocytes and treated with tunicamycin, an ER stress inducer. ER stress stimulation was confirmed by the upregulation of ER stress markers: binding immunoglobulin protein (*Bip*) and CCAAT-enhancer-binding protein homologous protein (*Chop*) (Figure 1A). Consequently, *Ucp1* expression was severely suppressed (Figure 1A). The addition of chemical chaperone 4-phenylbutyrate (PBA) alleviated ER stress markers *Bip* and *Chop*, while also rescued *Ucp1* expression (Figure 1A). These results indicate that ER stress suppressed *Ucp1* expression in beige adipocytes.

Accumulating evidence has identified the cross-talk between UPR and the mitogen-activated protein kinase (MAPK) signaling pathway as a result of ER stress stimulation [13]. Indeed, we found extracellular signal-regulated kinase (ERK) and c-Jun N-terminal kinase (JNK), which are parts of the MAPK signaling pathway, were activated by tunicamycin treatment in our experimental condition. Both ERK (Figure 1B) and JNK (Figure 1C) were phosphorylated after ER stress stimulation. The addition of either ERK or JNK inhibitor (U0126 or SP600125, respectively) could ameliorate tunicamycin-induced suppression of *Ucp1* expression (Figure 1D,E and Figure S1A,B) while inhibiting the phosphorylation of ERK (Figure 1F and Figure S1C) or JNK (Figure 1G and Figure S1D), indicating an ER stress-induced suppression of *Ucp1* expression via the activation of ERK and JNK pathway.

### 2.2. ER Stress Induces Downregulation of the Ucp1 Activator, Pparγ, Preferably via the JNK Pathway

**Figure 2:**
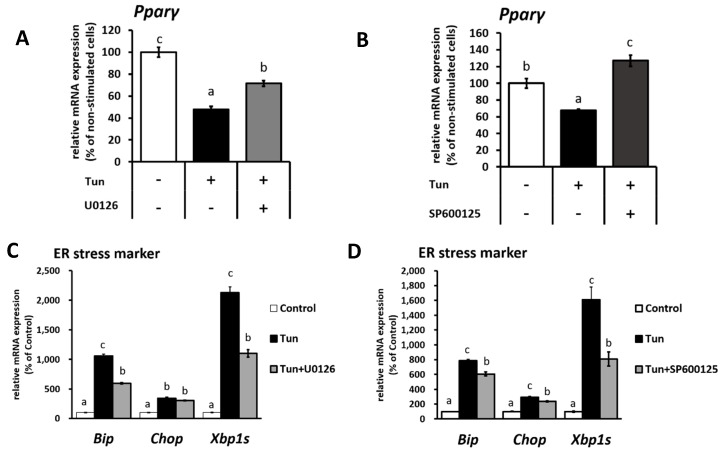
ER stress decreases *Ucp1* activator, peroxisome proliferator-activated receptor γ (*Pparγ*) mRNA expression through ERK and JNK pathway in IWAT cells. *Ppar*γ and ER stress marker (*Bip*, *Chop*, and X-box binding protein 1 splicing-*Xbp1s*) mRNA expression in cells treated with (**A**,**C**) 100 μM ERK inhibitor (U0126) for 14 h or (**B**,**D**) 25 μM JNK inhibitor (SP600125) for 13 h, followed by tunicamycin (1 μM, 12 h). Data are presented as mean ± S.E.M. (error bars). *n* = 4 in each group. Different letters indicate significant differences (*p* < 0.05) according to one-way ANOVA followed by Tukey–Kramer multiple comparison test.

It has been previously shown that Pparγ is a direct target of both ERK and JNK, which may result in decreased Pparγ transcriptional activity [28] and thus possibly affect *Ucp1* expression. Alongside *Ucp1* downregulation, the level of *Pparγ* mRNA was decreased upon tunicamycin treatment (Figure 2A,B) in IWAT cells but rescued when cells were treated with either an ERK (Figure 2A) or JNK inhibitor (Figure 2B). Besides that, ER stress markers (*Bip*, *Chop* and X-box binding protein 1 splicing (*Xbp1s*)) were partially recovered through ERK and JNK inhibition (Figure 2C,D), indicating the involvement of ERK/JNK pathway in regulating *Pparγ* expression.

**Figure 3:**
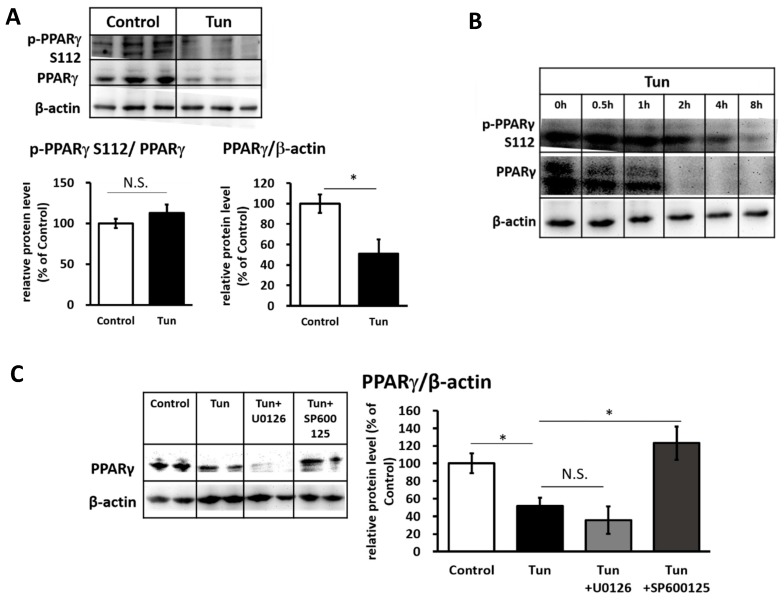
ER stress decreases Pparγ protein level preferably through JNK pathway in IWAT cells. Pparγ phosphorylation at serine 112 (S112) and Pparγ protein level in cells treated with 1 μM tunicamycin (**A**) for 12 h or (**B**) at different time points. (**C**) Pparγ protein after treated with 100 μM U0126 for 14 h or 25 μM SP600125 for 13 h, followed by tunicamycin (1 μM, 12 h). β-actin was used as a loading control. Data are presented as mean ± S.E.M. (error bars). *n* = 3–4 in each group. * indicates significant differences (*p* < 0.05) according to unpaired-*t* test. N.S., not significant.

We further investigated the possibility of post-translational modifications of Pparγ through the phosphorylation in serine 112 (S112), which is one of the main targets of ERK/JNK [29]. However, we did not find any change in the level of phosphorylation; instead, the Pparγ protein level was significantly reduced (Figure 3A). A time-course experiment revealed that the decrease in the Pparγ protein level was initiated earlier than the decrease in phosphorylation of Pparγ at S112 (Figure 3B). At this time (~4 h), ERK and JNK were both activated (Figure 1B,C). However, only treatment with the JNK inhibitor (SP600125), but not the ERK inhibitor (U0126), could rescue Pparγ protein levels (Figure 3C), suggesting that JNK is the main pathway for the tunicamycin-induced reduction in Pparγ protein.

**Figure 4:**
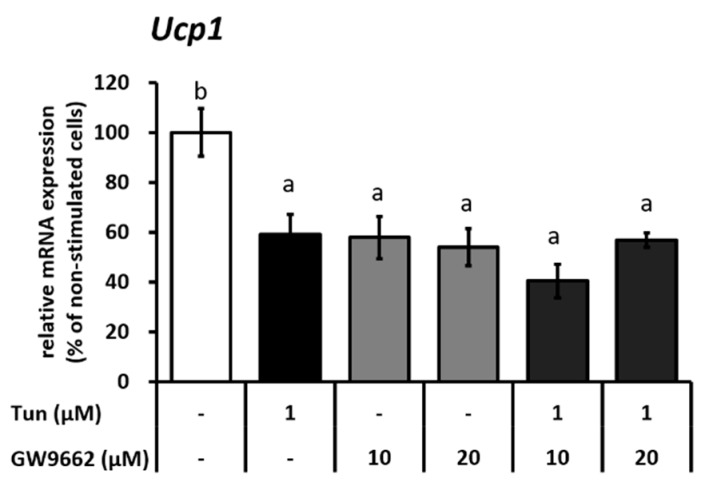
ER stress suppresses *Ucp1* expression through the reduced activity of Pparγ in IWAT cells. *Ppar*γ mRNA expression in cells treated with *Ucp1* mRNA expression after treatment with 10 or 20 μM Pparγ antagonist (GW9662) for 13 h, followed by 1 μM tunicamycin (12 h). Data are presented as mean ± S.E.M. (error bars). *n* = 4 in each group. Different letters indicate significant differences (*p* < 0.05) according to one-way ANOVA followed by Tukey–Kramer multiple comparison test.

Next, to confirm the role of Pparγ in ER stress-regulated *Ucp1* expression, we treated IWAT cells with Pparγ antagonist (GW9662). GW9662 similarly decreased *Ucp1* expression as tunicamycin (Figure 4 and Figure S2). However, the addition of GW9662 in tunicamycin treatment did not enhance the suppression of *Ucp1* expression (Figure 4 and Figure S2), suggesting that ER stress-suppressed *Ucp1* expression is indeed mediated through Pparγ. 

### 2.3. ER Stress Stimulates Pparγ Degradation that Leads to the Reduced Binding Activity

**Figure 5:**
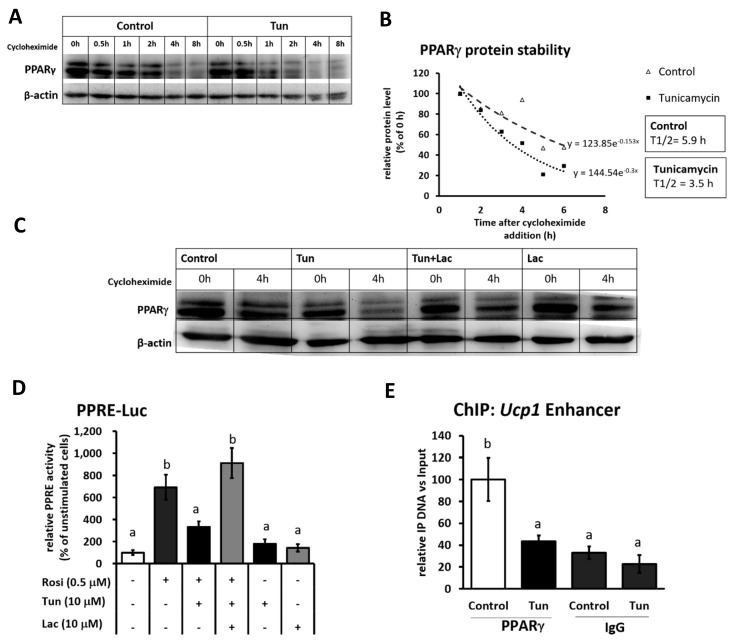
ER stress induces Pparγ degradation and reduces its binding activity in IWAT cells. (**A**) Pparγ protein level in cells during the cycloheximide chase experiment (20 μg/mL) at different time points with or without 1 μM tunicamycin treatment. (**B**) Regression analysis of Pparγ protein stability of (**A**). (**C**) Pparγ protein level after treated with 1 μM tunicamycin and/or 10 μM proteasome inhibitor, lactacystin (Lac) for 4 h in the presence of cycloheximide (20 μg/mL). β-actin was used as a loading control. (**D**) PPAR response element (PPRE) transcriptional activity in undifferentiated IWAT cells after treated with 0.5 μM rosiglitazone (Rosi) for 24 h, 10 μM tunicamycin and/or 10 μM lactacystin for 4 h, based on a luciferase assay. (**E**) Pparγ recruitment level in the *Ucp1* distal enhancer region analyzed by chromatin immunoprecipitation (ChIP) assay after cells were treated with 1 μM tunicamycin. IgG was used as a mock control. Data are presented as mean ± S.E.M. (error bars). *n* = 3–5 in each group. Different letters indicate significant differences (*p* < 0.05) according to one-way ANOVA followed by Tukey–Kramer multiple comparison test.

Next, to investigate whether the decreased Pparγ protein was dependent on the decrease in its mRNA expression, we performed a cycloheximide chase experiment to measure Pparγ protein stability. Tunicamycin treatment seemed to accelerate Pparγ degradation under cycloheximide addition (Figure 5A). The half-life of Pparγ was significantly reduced from 5.9 to 3.5 h upon tunicamycin treatment (Figure 5B). However, the addition of proteasome inhibitor (lactacystin) could rescue Pparγ protein (Figure 5C), suggesting that the degradation of Pparγ was mediated via proteasome degradation.

To further examine the consequence of Pparγ degradation for its role as activator, luciferase assay was done to analyze PPAR response element (PPRE) transcriptional activity under tunicamycin stimulation. Although it was in undifferentiated IWAT cells, a luciferase assay clearly showed that the tunicamycin treatment canceled the upregulation of PPRE transcriptional activity by rosiglitazone (Figure 5D). However, the stabilization of Pparγ by lactacystin altered PPRE activity (Figure 5D), indicating that Pparγ degradation indeed affects its binding activity. To finally connect the decrease in Pparγ binding activity to *Ucp1* expression, we performed chromatin immunoprecipitation (ChIP) assay to measure the recruitment of Pparγ onto the PPRE within the *Ucp1* enhancer region. As shown in Figure 5E, the recruitment level of Pparγ to *Ucp1* promoter was significantly decreased after tunicamycin treatment. These data established that ER stress-induced Pparγ degradation was associated with the downregulated *Ucp1* expression due to the loss of Pparγ activator binding to *Ucp1* promoter.

### 2.4. ER Stress Suppressed Both Ucp1 mRNA and Protein Expression in Adipose Tissue

**Figure 6:**
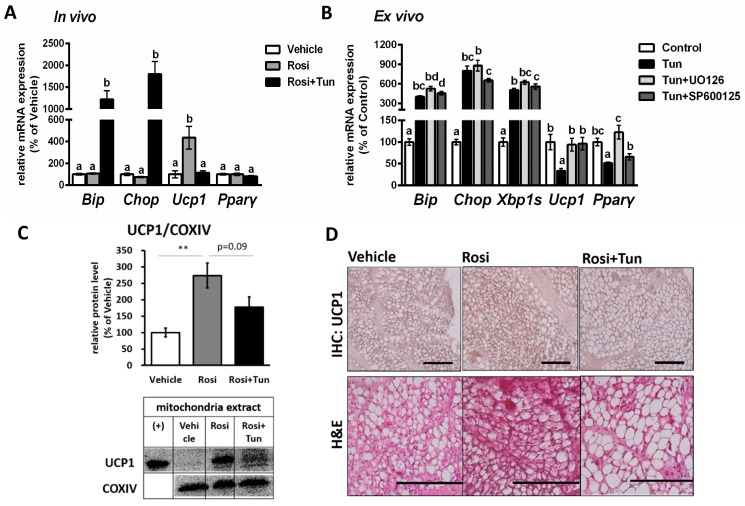
ER stress stimulation negatively regulates *Ucp1* expression in IWAT in vivo and ex vivo. (**A**) The mRNA expression levels of ER stress markers *Bip*, *Chop*, and *Xbp1s*, and adipocytes browning markers *Ucp1* and *Pparγ* in (**A**) IWAT in vivo of mice injected with vehicle, 10 mg/kg rosiglitazone for 10 days (Rosi), or rosiglitazone and 10 mg/kg tunicamycin (Rosi+Tun) for the last 24 h or (**B**) IWAT ex vivo after treated with 1 μM tunicamycin for 24 h with or without 100 μM U0126 for 26 h and 25 μM SP600125 for 25 h. (**C**) Ucp1 protein level, and (**D**) immunohistochemical staining (IHC) of Ucp1 (scale bar 200 μM) as well as hematoxylin and eosin (H&E) staining (scale bar 500 μM) in IWAT in vivo. (+) indicates the positive control (brown adipose tissue). Cytochrome c oxidase subunit IV (COXIV) were used as loading controls. Data are presented as mean ± S.E.M. (error bars). *n* = 3–8 in each group. Different letters indicate significant differences (*p* < 0.05) according to one-way ANOVA followed by Tukey–Kramer multiple comparison test. ** indicates significant differences (*p* < 0.01) according to unpaired-*t* test. N.S., not significant.

After observing a negative regulation of ER stress on *Ucp1* expression in vitro, we next investigated if the same phenomena also occur in vivo. Browning of WAT was induced by injecting Pparγ agonist (rosiglitazone) for 10 days. As shown by the expression level of ER stress marker genes *Bip* and *Chop* (Figure 6A), the rosiglitazone treatment had no effect on ER stress. IWAT, known as the most typical browning-stimulated WAT, was confirmed to undergo browning through a significant increase in *Ucp1* mRNA level (Figure 6A) after rosiglitazone treatment. After beige adipocytes were developed in IWAT, ER stress was afflicted through tunicamycin treatment. A single injection of tunicamycin could stimulate ER stress in IWAT as indicated by elevated *Bip* and *Chop* (Figure 6A) mRNA levels. In this state, tunicamycin canceled *Ucp1* mRNA upregulation by rosiglitazone to basal levels (Figure 6A). However, similar to in vitro result, ERK and JNK inhibition could ameliorate the tunicamycin-suppressed *Ucp1* expression ex vivo (Figure 6B). It is important to note that ERK and JNK inhibition barely altered ER stress marker (*Bip*, *Chop*, and *Xbp1s*) (Figure 6B), suggesting that the alteration of ERK and JNK pathway possibly only affect the downstream signaling of UPR, which in this case was *Ucp1* regulation.

**Figure 7:**
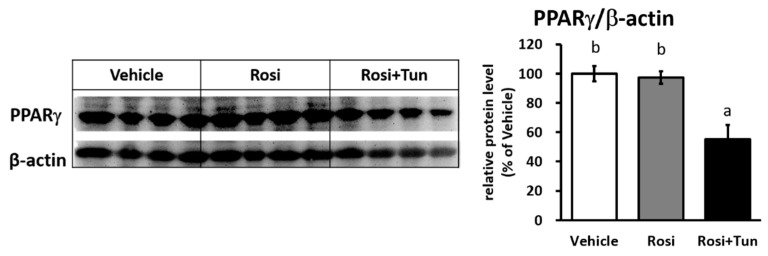
ER stress reduced Pparγ protein in IWAT. Pparγ protein level in IWAT of mice injected with vehicle, 10 mg/kg rosiglitazone for 10 days (Rosi), or rosiglitazone and 10 mg/kg tunicamycin (Rosi+Tun) for the last 24 h. β-actin were used as loading controls. Data are presented as mean ± S.E.M. (error bars). *n* = 3–8 in each group. Different letters indicate significant differences (*p* < 0.05) according to one-way ANOVA followed by Tukey–Kramer multiple comparison test.

As mRNA expression was reduced, the Ucp1 protein level was subsequently decreased under ER stress stimulation (Figure 6C and Figure S3A). The Ucp1 downregulation was also observed via immunohistochemistry (IHC) of Ucp1 (Figure 6D) in IWAT. Hematoxylin and eosin (H&E) staining showed an increasing size of lipid droplet after tunicamycin injection (Figure 6D and Figure S4), indicating a reversal of browning (whitening). These data demonstrated that ER stress is a strong negative regulator of Ucp1 expression in IWAT. Following the in vitro result, we also found a significant reduction in Pparγ protein levels in vivo (Figure 7 and Figure S3B), but not mRNA levels (Figure 6A), suggesting the decreased Pparγ protein as the main factor for *Ucp1* downregulation during ER stress stimulation.

## 3. Discussion

ER stress and inflammation have been known as a hallmark of metabolic syndrome. While how inflammation could defect Ucp1 expression in adipose tissue has been reported [23,30], the effect of ER stress in Ucp1 regulation is still unclear. However, as shown in Figure 6C, Ucp1 protein level was not detected in the basal level of IWAT (without rosiglitazone). Subsequently, Ucp1 protein level remained undetected when ER stress was induced by tunicamycin (Figure S3A). Therefore, we decided to investigate the effect of ER stress specifically in beige adipocytes. We first needed to induce the formation of beige adipocytes in IWAT by using drug-induced BAT activation. Among those, β3-adrenergic receptor agonist (such as isoproterenol) and the activators of Pparγ (such as rosiglitazone) have been widely used for treating obesity and type 2 diabetes through the browning-related modification in carbohydrate and lipid metabolism [31,32]. Isoproterenol and rosiglitazone themselves have been reported to increase the levels of *Ucp1* mRNA in adipocytes, thus both drugs can be used to induce beige adipocytes [31,33,34,35]. However, isoproterenol is reported to stimulate ER stress [36] while activating *Ucp1*, as confirmed in our preliminary experiment (data not shown), therefore using isoproterenol did not fit this study. Unlike isoproterenol, rosiglitazone could induce browning in beige adipocytes without afflicting ER stress, which is shown in this report. Hence, the use of rosiglitazone would not hinder the main ER stress stimulator from tunicamycin. It is also important to note that the long treatment of rosiglitazone has been reported to have adverse effects such as weight gain (due to fluid retention) and the expansion of adipose tissue in many studies [37]. However, we have confirmed that our experimental model was done with no side effect in body weight (data not shown) or adipose tissue expansion (Figure 6D).

Pparγ has been widely known as an important activator that could influence *Ucp1* expression [1,38,39]. Interestingly, we found that Pparγ protein level is decreased under ER stress stimulation even at the basal level ( s3 B). Thus, there is a possibility that ER stress-reduced Pparγ protein would further affect Ucp1 expression and prevent WAT to undergo browning. Although this possibility could not be proven due to low Ucp1 detection in WAT, our study clearly shows that ER stress was indeed a strong negative regulator of both *Ucp1* mRNA and protein expression in beige adipocytes. Furthermore, ER stress stimulation seemed to increase the lipid droplet size even in the activated beige adipocytes, which indicates whitening (reversal of browning) as shown in this study, suggesting ER stress may closely involve to the impaired browning.

To find the mechanism under ER stress-suppressed *Ucp1* expression, we first noticed the major involvement of MAPK pathways: ERK and JNK as a part of UPR during ER stress. Among the three ER stress sensors that have been established, IRE1 activation has been reported to promote the phosphorylation of ERK and JNK [13,20,40], which was also shown in this study. However, the consequences of ERK and JNK activation to *Ucp1* expression during ER stress is unknown. Herein, we found that both ERK and JNK inhibition could recover *Ucp1*_expression under ER stress condition, suggesting that ER stress suppressed-*Ucp1* expression might be mediated through ERK and JNK pathways. Furthermore, *Pparγ* regulation by ERK and JNK during ER stress stimulation seemed to have similar pattern as *Ucp1*. Following that, Pparγ protein level was also decreased by tunicamycin treatment. However, only JNK inhibition could rescue Pparγ protein level. These results suggesting that the reduced *Ucp1* expression was mediated preferably through JNK-Pparγ interaction.

It has been known that Pparγ is a phosphorylation target of MAPK which includes ERK and JNK [29]. Pparγ phosphorylation at Serine 112 by either ERK or JNK has been known to change Pparγ conformation that lead to the protein degradation and repressed transcriptional activation [29,41]. However, to our surprise, we did not find significant change in phosphorylation of Pparγ. Instead, Pparγ protein level was decreased earlier than its phosphorylation. Thus, we assume that although JNK-mediated the reduction of Pparγ activity by phosphorylation has been established through several studies [29,41,42], the detail mechanism may be different depending on the source of JNK activation. In the case of ER stress, JNK might mediate the decrease of *Pparγ* mRNA and protein level independent of phosphorylation modification. Accordingly, JNK activation is also known to be associated with apoptosis through promoting phosphorylation and activation of pro-apoptotic protein Bcl-2-associated X protein (Bax) [13,43,44]. Indeed, we found that chronic ER stress (12–20 h) in our experimental condition induced cell apoptosis, indicated by the upregulated *Chop* expression, decreased cell viability (Figure S5A), and increased in cleavage Caspase 3 (Figure S5B,C). *Chop* has been reported to participate in cell death program during ER stress [45]. On the other hand, Caspase 3 (apoptosis marker) has been found to mediate Pparγ cleavage [46]. These pieces of evidence suggest that JNK-mediated reduced Pparγ activity may be related to the cell apoptosis response.

Regardless, we found a consistent decrease in Pparγ activity in vitro, ex vivo, and in vivo after ER stress stimulation. Indeed, when we reduced Pparγ activity by Pparγ antagonist (GW9662), it similarly downregulated *Ucp1* expression as tunicamycin. Furthermore, the co-treatment between tunicamycin and GW9662 did not enhance the suppression of *Ucp1* expression, suggesting that both compounds regulated *Ucp1* expression preferably through the same pathway, which in this case was through Pparγ. To finally connect the consequence of Pparγ reduction to *Ucp1* expression, we investigated the PPRE activity under tunicamycin treatment. *Ucp1* is one of the established activation target of Pparγ in adipose tissue, equipped with PPRE binding sequence located on the *Ucp1* distal enhancer (-2494 to -2318 bp) [1]. Herein, we showed that PPRE activity was significantly affected after ER stress stimulation, based on the luciferase assay. Although it was performed in undifferentiated IWAT cells due to the low transfection efficiency in differentiated cells, we further confirmed in ChIP assay that Pparγ recruitment to *Ucp1* promoter was consistently reduced in differentiated IWAT cells. These pieces of evidence thus show that the reduced Pparγ binding to *Ucp1* promoter are likely due to the decreased protein level and subsequently affected *Ucp1* mRNA downregulation as ER stress was stimulated.

While the reduced *Pparγ* mRNA expression was only seen in vitro, the Pparγ protein level was decreased both in vitro and in vivo. These results might indicate that the loss of Pparγ protein was not dependent on its mRNA expression. Later, we found that Pparγ protein stability was disturbed by ER stress stimulation, suggesting that Pparγ protein was degraded. In addition, it has been known that ER stress induction could lead to the activation of protein degradation pathway to remove the accumulation of unfolded protein [14]. Basically, there are two major protein degradation pathways: proteasomes (via ER-associated degradation or ERAD) and lysosome (via autophagy) degradation [14,47]. Between two pathways, ERAD is recognized as the predominant cellular mechanism for removal of unfolded protein [14,48]. Certainly, the addition of proteasome inhibitor (lactacystin) rescued Pparγ protein and PPRE activity, suggesting proteasome-mediated Pparγ degradation affects its binding activity to *Ucp1* promoter during ER stress stimulation in beige adipocytes.

Interestingly, the induction of ER stress did not only affect *Ucp1* mRNA expression, but also Ucp1 protein level. Although, it might be linear to the decreased in mRNA expression, there still has possibility that the reduced Ucp1 protein level was affected directly through ERAD mechanism. Therefore, further investigation is needed to carefully assess Ucp1 protein modification in ER stress. Regardless, this study presents a novel evidence about the negative regulation of ER stress signaling to *Ucp1* expression via the suppression of activator Pparγ in beige adipocytes. 

## 4. Materials and Methods

### 4.1. Materials

All chemicals were obtained from Nacalai Tesque (Kyoto, Japan), Wako (Osaka, Japan), Corning (Corning, NY, USA), Qiagen (Hilden, Germany), Invitrogen (Carlsbad, CA, USA), and Sigma-Aldrich (St. Louis, MO, USA). Tunicamycin, U0126, SP600125, and cycloheximide were purchased from Nacalai Tesque (Kyoto, Japan). GW9662 (Sigma-Aldrich, St. Louis, MO, USA), rosiglitazone (LKT Laboratories, St. Paul, MN, USA), and PBA (Santa Cruz Biotechnology, Dallas, TX, USA) were acquired from the indicated companies.

### 4.2. Animal Experiment

Mice were kept in a temperature-controlled room at 23 ± 1 °C with a 12 h light/dark cycle and free access to food (standard diet) and water. To stimulate browning, 6–10-week-old male C57BL/6J mice (SLC, Shizuoka, Japan) were intraperitoneally injected with 10 mg/kg rosiglitazone daily for 10 days [49]. On Day 10, 10 mg/kg tunicamycin was injected to induce ER stress. Mice were then fasted overnight. Twenty-four hours after the last injection, mice were sacrificed, and IWAT was harvested for mRNA and protein analysis. The mice were handled in accordance with procedures approved by the Kyoto University Animal Care Committee (Permission number: 29-62, 20 April 2012).

### 4.3. Ex Vivo Experiment

To stimulate browning, 6-week-old male C57BL/6J mice (SLC, Shizuoka, Japan) were intraperitoneally injected with 10 mg/kg rosiglitazone daily for 10 days [49]. Twenty-four hours after last injection, IWAT was collected and immediately incubated in serum free medium containing 1 μM tunicamycin for 24 h with or without 100 μM ERK inhibitor (U0126) for 26 h/25 μM JNK inhibitor (SP600125) for 25 h. IWAT was then extracted for mRNA analysis.

### 4.4. Cell Culture

Immortalized primary IWAT cells were kindly provided by Dr. S. Kajimura (University of California, San Francisco, CA, USA). IWAT cells were maintained in a humidified 5% CO_2_ atmosphere at 37 °C using basic medium (DMEM) supplemented with 10% fetal bovine serum and 1% penicillin/streptomycin. To induce the differentiation into beige adipocytes [49], two days post-confluent IWAT cells were stimulated with 0.5 mM 1-methyl-3-isobutylxanthine, 2 μg/mL dexamethasone, 10 μg/mL insulin, 1 nM triiodo-L-thyronine (T3), 0.5 μM rosiglitazone (rosi), and 125 μM indomethacin for 48 h. The media was then replaced by basic medium containing 5 μg/mL insulin, 1 nM T3, and 0.5 μM rosi every 2 days. Generally, the differentiation process took 8–10 days. ER stress was induced by the addition of tunicamycin (1 μM) in serum-free medium for 12 h, unless mentioned.

### 4.5. RNA Preparation and Quantification of Gene Expression

RNA was extracted as described previously [50]. Total RNA was collected from cultured cells or tissues using Sepasol-RNA I Super G (Nacalai Tesque, Kyoto, Japan) or QIAzol Lysis Reagent (Qiagen, Hilden, Germany), respectively. RNA expression was quantified by real-time PCR using a LightCycler System (Roche Diagnostics, Mannheim, Germany) with SYBR green fluorescence signal detection. All mRNA signals were normalized to a *36b4* internal control. The primer sequences are listed in Table 1.

### 4.6. Protein Extraction and Western Blotting

Western blotting was performed as previously described [50]. The protein concentration was measured using the DC protein assay (Bio-Rad, Hercules, CA, USA). The primary antibodies included anti-Ucp1 (Sigma-Aldrich, St. Louis, MO, USA), anti-COXIV, anti-phospho-p44/42 MAPK (ERK1/2), anti-phospho-SAPK/JNK (Thr183/Tyr185), anti-SAPK/JNK, anti-Pparγ, and anti-β-actin (all purchased from Cell Signaling Technology, Danvers, MA, USA). The secondary antibody staining was visualized using a chemiluminescent horseradish peroxidase substrate (Millipore, Burlington, MA USA).

### 4.7. Chromatin Immunoprecipitation (ChIP) Assay

The ChIP assay was performed as described previously [50]. The cells were subjected to overnight immunoprecipitation with 8 μg Pparγ antibody (Perseus Proteomics, Tokyo, Japan), or rabbit IgG isotype control (Novus Biological, Littleton, CO, USA) as a mock control and analyzed by real-time PCR. The primer sequences are listed in Table 2.

### 4.8. Luciferase Assay

The luciferase assay was performed according to company protocol (Invitrogen, Carlsbad, CA, USA). Undifferentiated IWAT cells were transfected with plasmid containing the reporter vector driven by PPAR response element (PPRE-Luc) and a Pparγ expression vector using Lipofectamine 2000 reagent (Invitrogen, Carlsbad, CA, USA). The transfected cells were treated with 0.5 μM rosiglitazone for 24 h and 10 μM tunicamycin with or without 10 μM lactacystin for 4 h.

### 4.9. Statistical Analysis

All data were analyzed using Student’s *t*-test or one-way ANOVA followed by Tukey–Kramer test, when variances were heterogeneous. All data are presented as means ± SEM. Differences were considered significant at *p* < 0.05.

## 5. Conclusions

The present study highlights a novel mechanism of ER stress regulation to *Ucp1* expression preferentially via the suppression of activator Pparγ in beige adipocytes. Furthermore, it may provide a solid foundation about the involvement of ER stress to the impaired thermogenic capacity, especially in obese adipose tissue. Thus, recovering ER stress is proven essential to rescue thermogenic activity in adipose tissue.

## Figures and Tables

**Table 1:** Primers used for RNA quantification.

Gene	Forward	Reverse
*Ucp1*	5′-CAAAGTCCGCCTTCAGATCC-3′	5′-AGCCGGCTGAGATCTTGTTT-3′
*Pparγ*	5′-GGAGATCTCCAGTGATATCGACCA-3′	5′-ACGGCTTCTACGGATCGAAAACT-3′
*36b4*	5′-TCCTTCTTCCAGGCTTTGGG-3′	5′-GACACCCTCCAGAAAGCGAG-3′
*Bip*	5′-GTTTGCTGAGGAAGACAAAAAGCTC-3′	5′-CACTTCCATAGAGTTTGCTGATAAT-3′
*Chop*	5′-GTCCAGCTGGGAGCTGGAAG-3′	5′-CTGACTGGAATCTGGAGAG-3′

**Table 2:** Primers used in ChIP assay.

Gene	Forward	Reverse
*Ucp1* enhancer	5′-CTCCTCTACAGCGTCACAGAGG-3′	5-AGTCTGAGGAAAGGGTTGA-3′

## Supplementary Materials

Supplementary Materials can be found at http://www.mdpi.com/1422-0067/20/2/274/s1. Figure S1: The effect of extracellular signal-regulated kinase (ERK) and c-Jun N-terminal kinase (JNK) inhibitor in beige adipocytes; Figure S2: The effect of peroxisome proliferator-activated receptor gamma (Pparγ) antagonist treatment in beige adipocytes; Figure S3: The effect of ER stress induction in IWAT; Figure S4: The lipid size of IWAT after ER stress stimulation; Figure S5: ER stress stimulation induces cell death program in beige adipocytes.

## Abbreviations

β-AR	beta-adrenergic receptor
BAT	brown adipose tissue
Bip	binding immunoglobulin protein
ChIP	chromatin immunoprecipitation
Chop	CCAAT-enhancer-binding protein homologous protein
COXIV	cytochrome c oxidase subunit IV
ER	endoplasmic reticulum
ERAD	ER-associated degradation
ERK	extracellular signal-regulated kinase
H&E	hematoxylin and eosin
IHC	immunohistochemical staining
IRE1α	inositol-requiring enzyme 1 alpha
IWAT	inguinal white adipose tissue
JNK	c-Jun N-terminal kinase
MAPK	mitogen-activated protein kinase
PBA	4-phenylbutyrate
Pparγ	peroxisome proliferator-activated receptor gamma
PPRE	PPAR response element
Rosi	rosiglitazone
S112	serine 112
T3	triiodo-L-thyronine
Ucp1	uncoupling protein 1
WAT	white adipose tissue
XBP1s	X-box binding protein 1 splicing
