# Supplementary material for: Endoplasmic Reticulum Stress Impaired Uncoupling Protein 1 Expression via the Suppression of Peroxisome Proliferator-Activated Receptor γ Binding Activity in Mice Beige Adipocytes
Source: Int J Mol Sci. 2019 Jan 11;20(2):274. doi: 10.3390/ijms20020274 (PMC6359291; doi:10.3390/ijms20020274)
Supplement: Supplementary file 1 [file ijms-20-00274-s001.pdf]

Supplementary Material

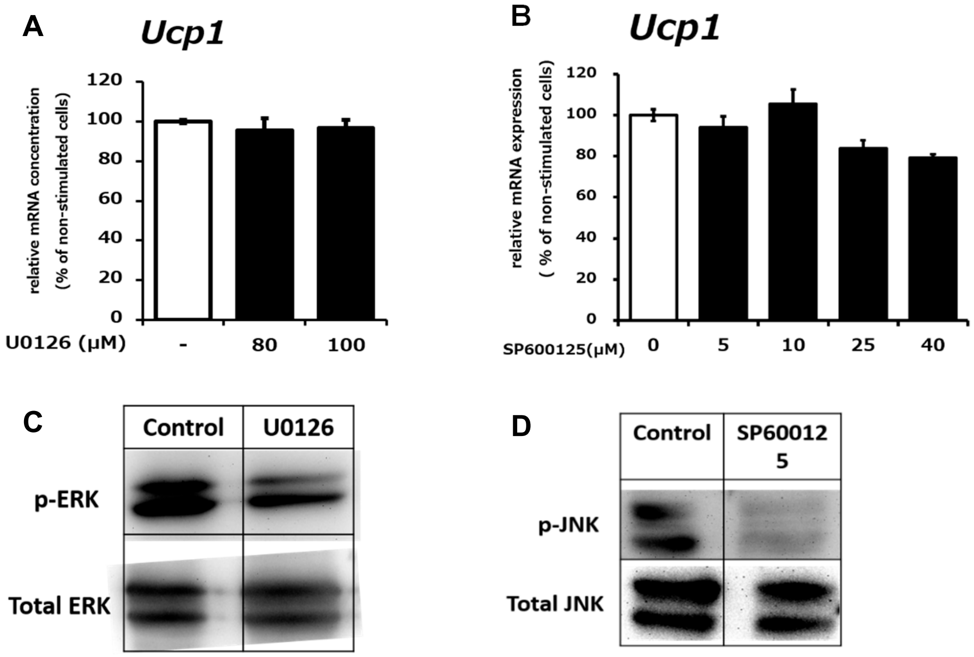

Figure S1. The effect of extracellular signal-regulated kinase (ERK) and c-Jun N-terminal kinase (JNK) inhibitor in beige adipocytes. Uncoupling protein 1 (*Ucp1*) mRNA expression in immortalized inguinal white adipose tissue cells (IWAT cells) after treated at different concentration of (A) ERK inhibitor (U0126) or (B) JNK inhibitor (SP600125) for 14 h or 13 h, respectively. The phosphorylation of (C) ERK or (D) JNK in IWAT cells after treated with 100 μM U0126 for 14 h or 25 μM SP600125 for 13 h, respectively. Data are presented as mean ± S.E.M. (error bars). n = 4 in each group.

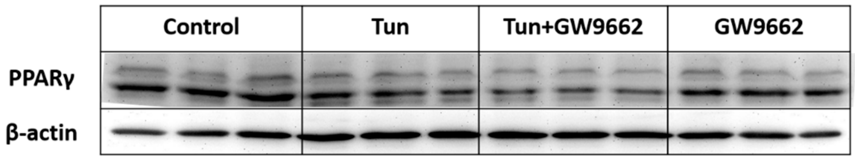

Figure S2. The effect of peroxisome proliferator-activated receptor gamma (Pparγ) antagonist treatment in beige adipocytes. Pparγ protein level in IWAT cells after treatment with 1 μM tunicamcyin for 12 h with or without 10 μM GW9662 for 13 h.

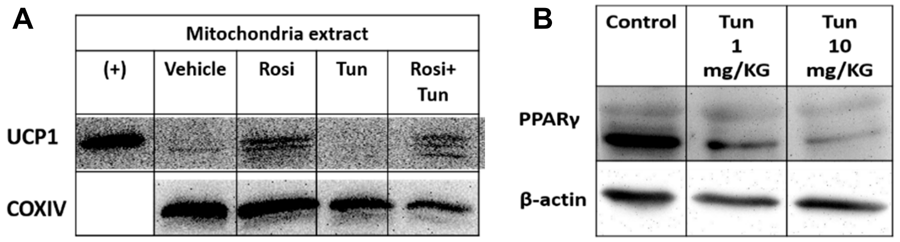

Figure S3. The effect of ER stress induction in IWAT. (A) UCP1 protein level in inguinal white adipose tissue (IWAT) of mice injected with vehicle, 10 mg/kg rosiglitazone for 10 days (Rosi), 10 mg/kg tunicamycin (Tun) for the last 24 h, or rosiglitazone and tunicamycin (Rosi+Tun) together. (B) Pparγ protein level in IWAT of mice injected with tunicamycin (dose: 1 mg/kg and 10 mg/kg) for 24 h. (+) indicates positive control (brown adipose tissue). Cytochrome c oxidase unite IV (COXIV) and β-actin were used as loading control.

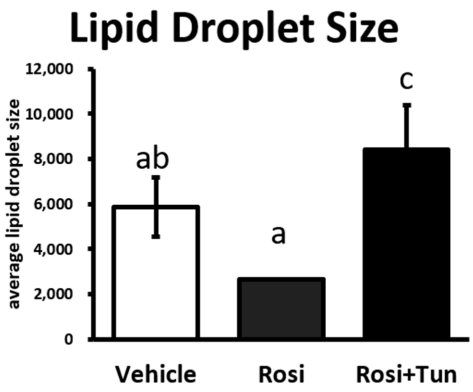

Figure S4. The lipid size of IWAT after ER stress stimulation. Lipid droplet size quantification of Figure 6D. Data are presented as mean ± S.E.M. (error bars). n = 65–130 in each group.

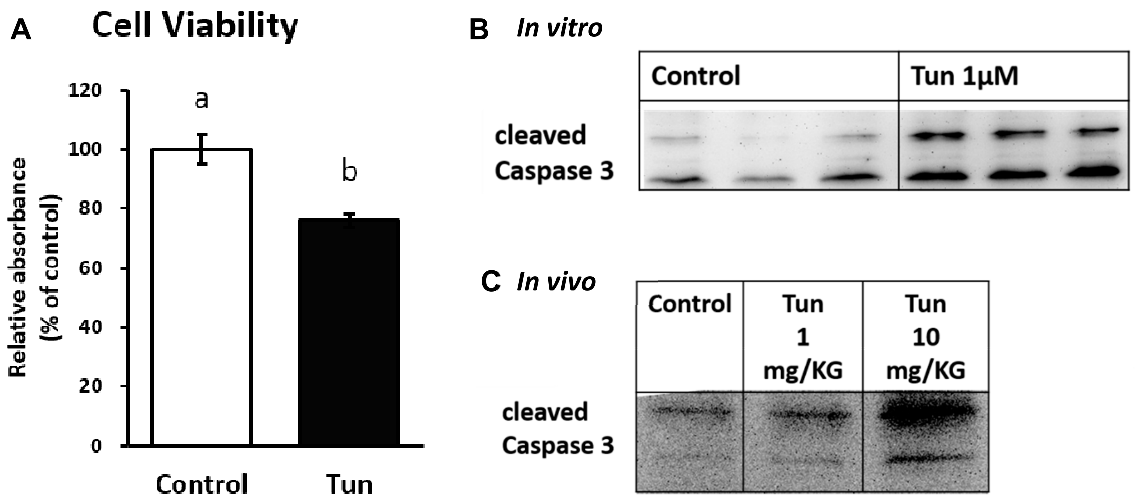

Figure S5. ER stress stimulation induces cell death program in beige adipocytes. (A) Cell viability of IWAT cells after treated with 1 μM tunicamycin for 12 h, analyzed by cell titer 96® Aqueous One Solution Cell Proliferation Assay (Promega, Madison, WI, USA). Cleaved caspase-3 protein level in (B) IWAT cells after treated with 1 μM tunicamcyin (Tun 1μM) for 12 h or (C) IWAT of mice injected with tunicamcyin (1 mg/kg or 10 mg/kg) for 24 h. Data are presented as mean ± S.E.M. (error bars). *n* = 5 each group.
